# Early and late cognitive and behavioral aspects associated with range use in free-range laying hens (*Gallus gallus domesticus*)

**DOI:** 10.1016/j.psj.2024.103813

**Published:** 2024-04-30

**Authors:** Vitor Hugo Bessa Ferreira, Jeanne Seressia, Nathalie Même, Jérémy Bernard, Marie-Hélène Pinard-van der Laan, Fanny Calenge, Alexandre Lecoeur, Louise Hedlund, Per Jensen, Vanessa Guesdon, Ludovic Calandreau

**Affiliations:** ⁎ CNRS, IFCE, INRAE, UMR PRC, Université de Tours, Nouzilly, France; † INRAE, PEAT, Nouzilly, France; ‡ INRAE, AgroParisTech, UMR GABI, Université Paris-Saclay, Jouy-en-Josas, France; § IFM Biology, AVIAN Behavioural Genomics and Physiology group, Linköping Universtiy, Linköping, Sweden; # JUNIA, Comportement Animal et Systèmes d'Elevage, Lille, France

**Keywords:** chicken, cognition, mood, temperament, welfare

## Abstract

Individual differences in free-range chicken systems are important factors influencing how birds use the range (or not), even if individuals are reared in the same environmental conditions. Here, we investigated how various aspects of the birds' behavioral and cognitive tendencies, including their optimism/pessimism, cognitive flexibility, sociability, and exploration levels, are associated with range use and how they may change over time (before and after range access). To achieve this, 100 White Leghorn laying hen chicks underwent three distinct behavioral/cognitive tests—the cognitive bias test, the detour test, and the multivariate test—prior to gaining access to the range, between 9 and 39 days of age. After range access was allowed (from day 71), birds' range use was evaluated over 7 nonconsecutive days (from 74–91 days of age). Subsequently, a subset of birds, classified as high rangers (n = 15) and low rangers (n = 15) based on their range use, underwent retesting on the same three previous tests between 94 and 108 days of age. Our results unveiled a negative correlation trend between birds' evaluation of the ambiguous cue and their subsequent range use (rho = -0.19, *p* = 0.07). Furthermore, low rangers were faster to learn the detour task (χ2 = 7.34, df = 1, *p* = 0.006), coupled with increased sociability during the multivariate test (rho = -0.23, *p* = 0.02), contrasting with their high-ranging counterparts, who displayed more exploratory behaviors (F[1,27] = 3.64, *p* = 0.06). These behavioral patterns fluctuated over time (before and after range access); however, conclusively attributing these changes to birds' aging and development or the access to the range remains challenging. Overall, our results corroborate that behavioral and cognitive individual differences may be linked to range use and offer novel perspectives on the early behavioral and cognitive traits that may be linked to range use. These findings may serve as a foundation for adapting environments to meet individual needs and improve animal welfare in the future.

## INTRODUCTION

As public awareness of animal welfare and animal production concerns continues to surge, there is a growing interest among both the general public and the scientific community in exploring alternative production systems. In contrast to conventional systems, where animals are typically reared indoors and under high density (i.e., animals per square meter), alternative systems, such as free-range ones, may provide animals with access to an outdoor area (i.e., the range) and the potential for greater choice over their own actions, enabling them to express a wider behavioral repertoire (Knierim, 2006; Campbell et al., 2017; Larsen et al., 2017; Sánchez-Casanova et al., 2020). This, in turn, is expected to lead to an improvement in animal welfare when compared to indoor systems (Knierim, 2006).

While free-range production presents some benefits for animals and is highly valued by consumers, it is not without its own set of challenges. In free-range chicken systems (including both laying hens and broiler chickens), for example, birds may face heightened risks of infectious and non-infectious diseases, thermal stress, and predation (Campbell et al., 2021; Bonnefous et al., 2022). Beyond these challenges, a concern in free-range chicken production revolves around the presence of within-flock inter-individual variability in the use of the range: chickens may exhibit varying levels of motivation to venture outside the barn and use the range. Notably, this variability in range use has been observed to lead to distinct zootechnical and welfare outcomes, even though individuals within a flock are raised under virtually identical conditions (Bari et al., 2020; Sibanda et al., 2020; Taylor et al., 2020; Wurtz et al., 2023). For example, free-range broiler chickens that made frequent visits further from the barn had lower body weight, but better gait scores and reduced corticosterone in response to handling and confinement, compared to their indoor-preferring counterparts (Taylor et al., 2020). Similarly, for free-range laying hens, high range use was associated with better plumage, fewer comb wounds, shorter nail length, higher spleen and gizzard weight, but lower body weight, fat, and muscle (Bari et al., 2020). Furthermore, outdoor preferring individuals were less fearful compared to indoor preferring ones during open-field tests (Campbell et al., 2016, 2019a; but see Wurtz et al., 2023 where contrasting findings on two laying hen hybrids indicate that while behavior and gastrointestinal variables correlated with the extent of range use, fear levels, and several clinical welfare indicators did not). Overall, these findings reveal that while animals are provided with the choice and opportunity to access a larger and enriched space—the range, presumed to improve their welfare—some may opt not to use it due to potential fearfulness and/or negative cognitive perceptions. This emphasizes the importance of understanding the individual behavioral and cognitive traits associated with range use. Such insights are crucial for comprehending the implications of these variations on individual health and overall welfare, and are essential for effectively adapting the environment to suit the needs of the animals.

In the quest for a better understanding of inter-individual variations in range use, some studies focused on the behavioral and cognitive individual traits that manifest prior to animals gaining access to the range. These studies seek to ascertain whether certain personality or temperament traits emerge during chicks’ early life and may serve as predictors of range use. Among broiler chickens, early behavioral indicators that correlated with range use primarily revolved around individuals’ sociability and foraging behaviors. Specifically, a negative correlation was observed between the willingness to approach conspecifics during a social motivation test and range use, while foraging behavior exhibited a positive correlation with range use (Ferreira et al., 2020a; Ferreira et al., 2021a). However, it is noteworthy that these findings did not consistently hold true across different stages of bird development and for different strains (Ferreira et al., 2022; Bonnefous et al., 2023). In the case of laying hens, a few studies investigated how birds’ exploratory and fear tendencies before range access may be related to range use. Using open field and maze tests, Campbell et al. (2021) revealed that some indicators of fear, such as the latency to eat in a maze and the latency to leave a start box when the maze contained a novel object, were negatively associated with subsequent ranging behavior (notably the number of range visits), while in a more recent study, the link between exploration, fearfulness and range use contrasts with previous results: pullets that spent less time near a novel object and crossed less zones during a novel test arena used the range more (Taylor et al., 2023). On the other hand, Kolakshyapati et al., (2020), examining older hens within a commercial setting and after range access, found that the time spent interacting with a novel object was associated with more frequent range use. These contradictions reveal that while each study may contribute to a better understanding on how and why animals interact with the range differently, they also raise new questions and point to the need for further in-depth longitudinal studies in this area.

Regarding cognitive factors, broiler chicks that exhibited greater range use later in life demonstrated a higher conditioned preference for a specific compartment within a two-compartment arena before range access: this preference was associated with the compartment where they needed to make some effort to find food (i.e., contra-freeloading), in contrast to the compartment where food was freely available (Ferreira et al., 2021a). For laying hens, it was shown that birds’ spatial abilities (or spatial cognition) before range access was positively correlated to range use (Campbell et al., 2018b, 2021). As a deeper understanding of chicken cognition and behavior is crucial for accurately assessing their welfare and gaining insights into how animals perceive and interact with their environment (ANSES, 2018), additional research is essential for developing individual solutions that meet the distinct needs of each flock member (or at least, most flock members) and promote greater range use (Ferreira et al., 2021; Degrande et al., 2023).

The main objective of this study was to advance our understanding of the intricate interplay between range use, behavioral traits, and cognition in free-range laying hens. Specifically, we aimed to investigate whether the behavioral and cognitive traits of chicks before gaining access to the range could be linked to their range use in later life. To address this, we employed 3 behavioral/cognitive tests to explore various facets that may or may not be connected to birds’ range use. These tests encompassed: 1) A cognitive bias test, to assess how birds perceive and interpret diverse environmental stimuli (positive, neutral, and negative ones); 2) A detour test, which aimed to test birds’ adaptability in response to changing situations; and finally, 3) A multivariate test, delving into various aspects of bird sociability, exploration, boldness, foraging, and their relationships to range use. Each of these assessments was conducted on the same individuals both before and after the birds gained access to the range to compare how these variables change over time. Building upon previous research, we hypothesized that an optimistic environmental perception, exploratory behaviors, especially foraging behavior, and spatial cognition would correlate positively with range use (Campbell et al., 2018a; Ferreira et al., 2021a; Hedlund et al., 2021; Ferreira et al., 2022; Taylor et al., 2023). Conversely, we anticipated a negative correlation between range use, cognitive flexibility and social motivation (Ferreira et al., 2020a; 2020c).

## MATERIAL AND METHODS

### Ethical Statement

This study was conducted at the Pôle d'Expérimentation Avicole de Tours (UE PEAT, INRAE, Experimental Poultry Facility, https://doi.org/10.15454/1.5572326250887292E12) of the INRAE Centre Val-de-Loire, France, from April to September 2023. It was conducted under the approval of the INRAE animal experimentation ethics committee, under the number project number APAFiS #41335-2023010515226780 v11, in compliance with the current French legislation.

### Animals and Housing

At 1-day-old, a total of 600 White Leghorn Novogen laying hen chicks (*Gallus gallus domesticus*) were introduced and housed together within a single barn (5 × 12 m, initial density of 10 birds/m^2^). Subsequently, this population was reduced to 450 birds when they reached 57 d of age (density of 7.5 birds/m^2^), as part of a separate phase of the project. Birds were monitored on a daily basis until they reached the age of 155 d when the experiment was over and the birds culled. Within the barn, birds had unrestricted access to both food (provided via eight circular plastic feeders with a diameter of 40 cm) and water (delivered through forty automatic nipple drinkers distributed across two 3 m rails). Additionally, they had access to perches (consisting of 4 ladder perches offering a total length of 7.5 m/perch) and nesting areas (comprising 8 nesting blocks, each containing 3 nest boxes measuring 32.5 × 41.6 × 41.8 cm). Bedding was provided in the form of wood shavings. The animals received different feed formulations adapted to their specific developmental stages to ensure their nutritional requirements were optimally met. Artificial lighting was continuously and uniformly provided (24 h of light) for the initial 6 d after placement. Subsequently, from d 7 to d 9, a 16-h lighting cycle was implemented, followed by an 8-hour lighting regimen from d 10 onwards. Natural light only entered the barn when ventilation hatches automatically opened to maintain constant humidity and temperature levels, as well as when pop-holes for range access were opened (from 71 d of age, between 8:30 am and 10:30 pm see below). The indoor ambient temperature was initially set at 33°C on the first day and gradually reduced until it reached 20°C degrees (minimum temperature kept until culling) when the birds were 35 d-old.

**Figure 1:**
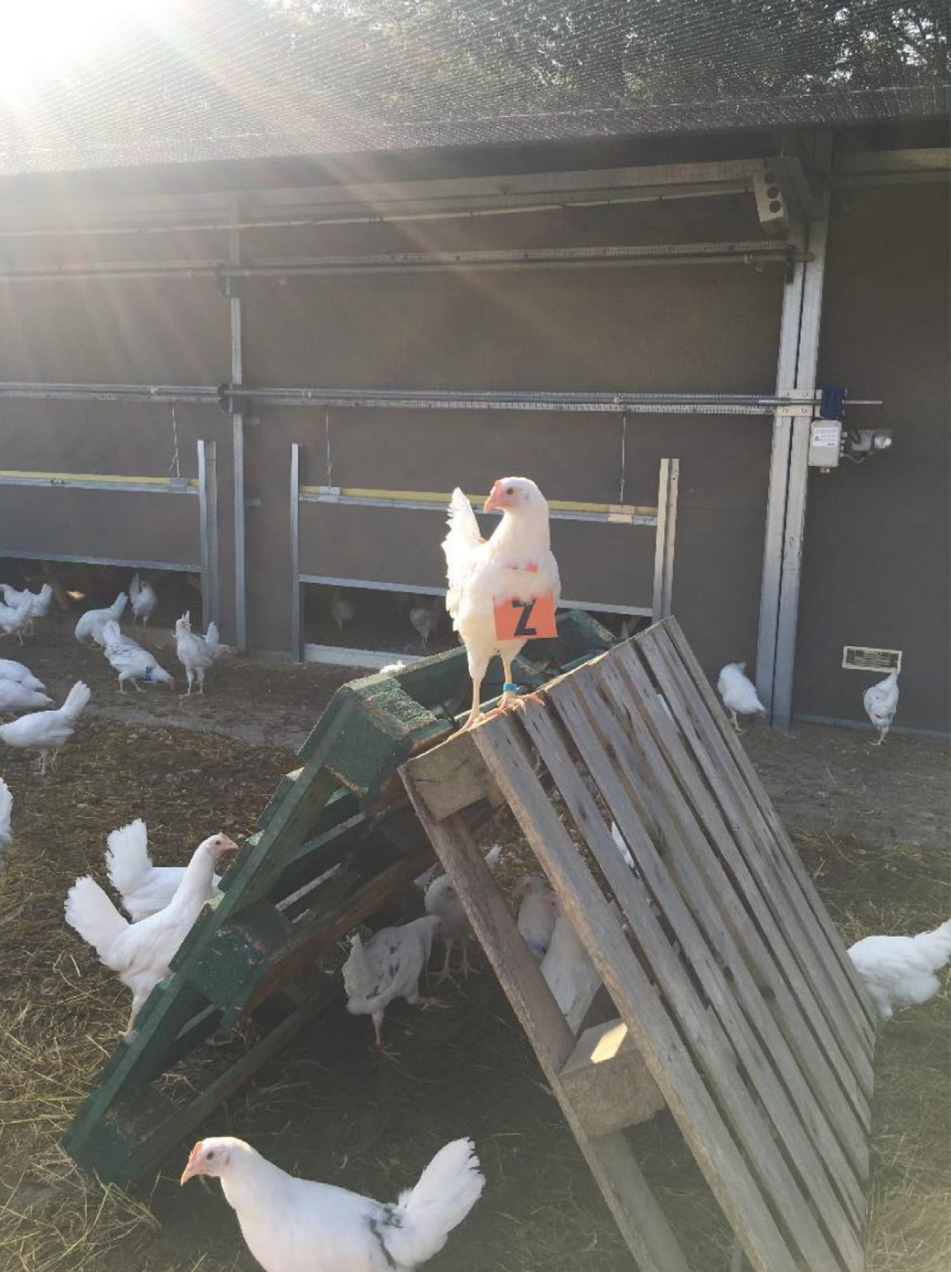
A pullet, over a shelter, on the range, carrying around her neck a brown poncho showing the letter Z. The poncho was a rectangular piece of plastic with unique acronyms for easy individual identification.

A subset of 100 chicks was randomly selected from the initial pool of 600 birds for participation in the behavioral and cognitive assessments. To distinguish these selected birds, they were assigned a unique wing tag bearing their individual identification number. Additionally, they were equipped with a colored ring on their legs: with an 8 mm ring at 8 d of age, a 10 mm ring at 30 d of age, and a 16 mm ring at 57 d of age. To facilitate easy identification without causing disruption to the birds, a nontoxic spray paint in 5 colors (orange, violet, rose, blue, and green - Raidex Animal Marking spray, Dettingen, Germany) was applied to the back of the chosen individuals. Furthermore, once their size allowed (at 57 d of age), distinctive rectangular colored plastic ponchos (10 colors - light orange, dark orange, yellow, pink, violet, green, blue, white, grey, and brown) each marked with unique acronyms, were placed around the birds’ necks. These ponchos were designed to adjust to the growth of the birds, with 2 different sizes employed: 13 × 5 cm from 57 to 70 d of age and 20 × 8.5 cm from 71 d of age onwards (Figure 1). This identification system not only guaranteed the easy recognition of each bird throughout the study but also minimized the time needed for individual capture and therefore reduced individual stress.

Starting at 71 d of age, all the individuals were granted access to an outdoor range measuring 45 × 12 meters. This access was facilitated through the opening of 4 pop-holes, each measuring 2 × 0.5 meters. The outdoor range consisted of a concrete area adjacent to the barn and a meadow-like open space with vegetation cover, providing uniform enrichment. This included 4 shelters and perches, as well as 4 shaded areas equipped with plastic sheeting measuring 2 × 2.5 meters. An antipredation net covered about 50% of the range to prevent aerial predation. All these elements were strategically positioned to encourage birds to make use of the range. The birds were allowed free access to the outdoor range between 8:30 am and 10:30 pm.

### Behavioral and Cognitive Tests and Ranging Behavior

All the behavioral and cognitive tests were carried out twice. The initial tests were carried out on a subset of 100 individuals between 9 to 39 d of age, to establish the behavioral and cognitive profiles of the birds at an early stage, before they were granted range access. Following the birds' access to the range starting from d 71 onwards, a new round of tests was conducted on a second subset of the 100 birds that had been previously tested. This second subset consisted of extreme users or non-users of the range, high rangers (birds that extensively used the range) and low rangers (birds that used the range only minimally). These latest tests took place between 94 and 108 d of age. See below for more information on range use measurements and bird classification.

Before range access, the cognitive bias and detour tests were administered over 5 consecutive days, with 20 chicks tested each day. The multivariate test was spread out over 3 consecutive days, during which 33 to 34 chicks were tested. Given that some of these tests heavily relied on the birds' social motivation, we prioritized the cognitive bias and detour tests as the initial assessments. This choice was made because social motivation is particularly pronounced in the early stages of life (McBride et al., 1969; Suarez and Gallup, 1983). The testing window for tests that were carried out after range access was condensed to 1 to 2 days.

In the morning, before starting each testing day, the animals were captured and placed in groups in wire-meshed waiting cages (1 × 0.5 × 0.5 m), located in the barn corridor. This placement ensured exposure to similar environmental conditions, such as lighting and temperature, as the non-tested individuals. During their time in the waiting cages (lasting between 1 and 10 h, depending on the duration of the test), the animals were visually isolated from the rest of the flock but could still interact through auditory cues. This was done for practical reasons and to minimize stress before the tests began. Throughout all the testing days, the birds had free access to food, water, wood shavings, and other tested conspecifics. Before the test, each individual, was gently caught from the waiting cage and taken to the testing room. Individuals were chosen in a predetermined pseudo-random order for tests conducted before range access. Following range access, we maintained balance by consistently alternating between testing a high ranger and a low ranger (and vice versa), ensuring an equitable distribution of testing times throughout the day. The testing room was maintained at an average temperature close to that of the barn at the moment of the test and was artificially illuminated at an average of 120 lx. The tests were conducted between 8:30 am and 6:30 pm. As soon as animals finished being tested for the day, they were released back into the barn.

**Figure 2:**
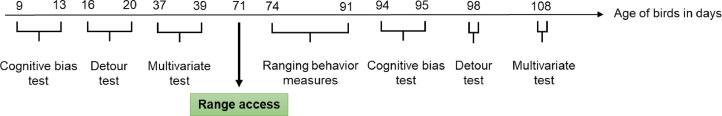
Schedule of the 3 behavioral and cognitive tests (cognitive bias test, detour test, and multivariate test) conducted on birds across two distinct periods: before and after range access. The first period spanned from 9 to 39 d of age, while the second period extended from 94 to 108 d of age. Range access was granted at 71 d of age, followed by 7 nonconsecutive days (between 74 and 91 d of age) of ranging behavior measurements. This process aimed to classify the 100 individuals studied into high rangers and low rangers based on their range use.

Finally, to investigate the links between the behavioral and cognitive variables assessed in the tests and each individual's ranging behavior, we conducted range use measurements over a period of 7 nonconsecutive days, distributed across 4 wk, spanning from 74 to 91 d of age. The timeline for behavioral/cognitive testing and ranging behavior measurements is outlined in Figure 2.

#### Cognitive Bias Test

The cognitive bias test drew inspiration from a methodology that had already been validated for assessing cognitive bias in domestic chicks (Salmeto et al., 2011; Hedlund et al., 2021, 2022). Our rationale for evaluating the cognitive bias of birds both before and after gaining access to the range stemmed from the understanding that the range, being a less predictable environment than the barn, requires birds to continually assess and respond to various stimuli they may encounter (Rodriguez-Aurrekoetxea et al., 2014; Campbell et al., 2018a; Ferreira et al., 2020b). Therefore, we hypothesized that birds that tend to assess stimuli in a more negative manner, essentially displaying a more pessimistic approach, could be less inclined to venture onto the range. Conversely, birds with a more optimistic approach to stimuli would be more willing to explore the range.

**Figure 3:**
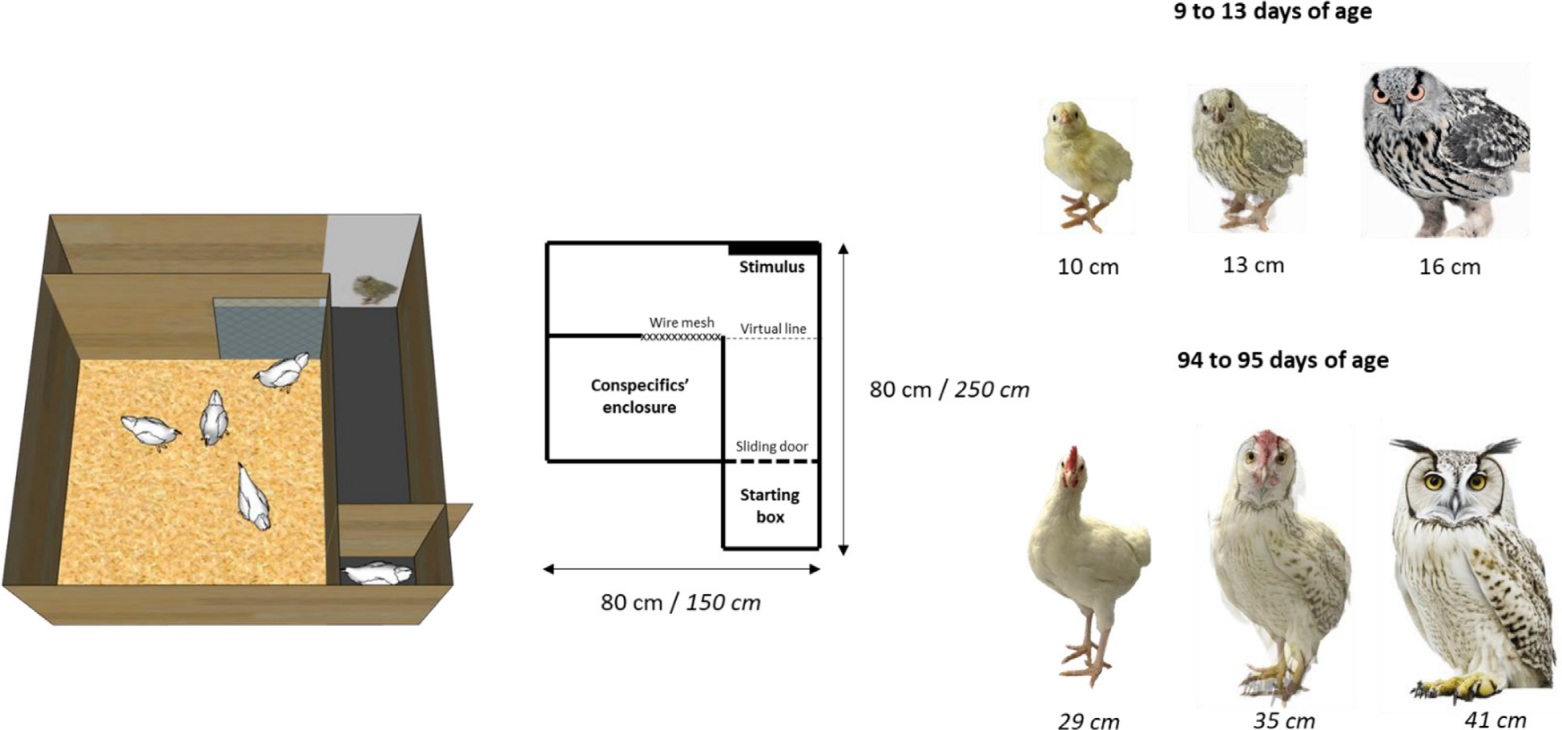
Schematic representation of the arenas used for testing cognitive bias in chicks (from 9 to 13 d of age), before range access, and pullets (from 94 to 95 d), after range access. The arena consists of a starting box connected to an L-shaped corridor, with the starting box at one end and an enclosure for conspecifics at the other end. Before reaching its conspecifics (visual contact was allowed through a wire mesh) and crossing the virtual line, the tested bird must pass close to different stimuli, presented once at each of the 4 trials: a mirror, a chick/pullet photo, a morph owl vs chick/pullet photo, and an owl photo. The stimuli were adapted to the age of the animals. Measurements in italics refer to the tests performed after range access.

The cognitive bias test was conducted when the animals were between 9 to 13 d of age (before range access) and then again between 94 to 95 d of age (after range access). The test was carried out in a straight alley maze (Salmeto et al., 2011), in a L-shaped arena. The tested animal was initially placed in a starting box located at one end of the corridor, while 4 conspecifics (randomly selected from individuals not participating in the study and changed every ten trials) were positioned at the opposite end of the corridor. These conspecifics served as a social reward for birds that successfully crossed the corridor. In the middle of the corridor, just before reaching the conspecifics' enclosure, the tested bird was exposed to 4 different stimuli that were visible from the starting box. These 4 stimuli included: 1) a mirror (representing the strongest positive stimulus and also as a control to assess if animals were willing to move within the arena), 2) a photo of a chick/pullet (representing a positive stimulus), 3) a photo of an owl (representing a negative stimulus), and 4) a photo that morphed the chick/hen and the owl (an ambiguous stimulus created using the program Morpheus Photo Morpher v.3.17 Standard). The photo of the chick/pullet was adjusted to match the size of the individuals being tested. To enhance the potentially frightening aspect of the stimuli, the sizes of both the ambiguous stimulus and the owl were systematically increased (Figure 3). All photos were printed in color against a white background.

The test started with the presentation of the mirror, which served as a highly positive stimulus. This initial step allowed birds to habituate to the testing environment and to increase their motivation to reach the social reward (i.e., the conspecifics at the end of the corridor). This stage also ensured that all individuals were sufficiently socially motivated and could proceed the testing with the other stimuli. Following the mirror stimulus, half of the animals were first exposed to the chick stimulus and then to the owl stimulus, while for the other half of individuals this presentation order was reversed (randomly assigned). Ultimately, all animals were confronted to the morph (i.e., the ambiguous stimulus).

For each stimulus, the test followed this structure: the tested individual was positioned in the starting box, and after a 10-second delay, a sliding door was opened. Subsequently, the bird had a 5-min window to explore the arena and reach its conspecifics by crossing a virtual line located at the end of the corridor, positioned at a distance of 15 cm from the stimulus. During each trial, the measured variable was the latency (in seconds) required for the individual to cross the virtual line (at least 50% of the animal's body passing the line). Animals that did not cross this line within 5 minutes when confronted with the mirror stimulus were permanently excluded from the test. In the case of the other stimuli, individuals who did not reach the virtual line within the initial 5-min period were then allocated a maximum time of 5 min (300 s). As soon as the individual crossed the virtual line, it was granted a few seconds of contact (via a wire mesh) with the conspecifics. Animals that did not cross the virtual line were gently touched from behind by the experimenter, encouraging them to move towards the social reward. Each individual had approximately 90 minutes in between subsequent trials. During this time individuals were placed in the waiting cages. The arena underwent thorough cleaning between each tested individual, with feces, feathers, and dust wiped away using a cloth.

#### Detour Test

The relationship between birds' spatial abilities, their ability to adapt to new situations, and their range use is a topic that is gaining continuous interest on free-range chicken research (Campbell et al., 2018a; Campbell et al., 2021; Ferreira et al., 2019, 2020a, 2020c; Taylor et al., 2023). However, current findings have not demonstrated consistent patterns across various studies. Research on broilers and laying hens, for example, has revealed disparities: outdoor-preferring broilers displayed lower spatial cognition and cognitive flexibility (Ferreira et al., 2019, 2020a, 2020c), while outdoor-preferring laying hens appeared to exhibit superior spatial cognition (Campbell et al., 2018b; Campbell et al., 2021) than their indoor-preferring counterparts. Additional investigation is needed to determine whether these differences arise from the distinct selective pressures experienced by broiler chickens and laying hens (Lindqvist et al., 2006), or if they are influenced by methodological disparities across studies. These could include variations such as conducting cognitive tests exclusively after range access or discrepancies between studies conducted on experimental vs. commercial conditions.

The detour test, along with its various adaptations, is a commonly employed method to assess animals' basic spatial abilities and their ability to adapt to new situations (Norman et al., 2019; Ferreira et al., 2020c). This test was conducted when the animals were between 16 to 20 d of age (before range access) and then again at 98 d of age (after range access). The test was conducted within a square wooden arena, featuring a U-shaped wooden starting point. The central wall of this starting point could either be opaque during the learning phase or a wire screen in the testing phase. This central wall either blocked or allowed the tested bird to observe 2 of its conspecifics situated on the opposite side of the arena in a compartment adjacent to the arena. Similar to the cognitive bias test, conspecifics were used as a social reward, and they were randomly selected from individuals not involved in the study. These conspecifics were changed every 5 tested individuals.

The test was structured into 2 distinct phases: the learning phase and the testing phase. During the learning phase, each bird initially spent 30 s in the same compartment as its conspecifics. Subsequently, the tested individual was placed at the starting point and was allotted a maximum of 1 min to detour around the opaque walls of the U-shaped zone, cross the virtual line and reach its conspecifics. If the individual failed to do so within this time frame, the experimenter would gently touch the bird from behind, encouraging and guiding it towards achieving the intended goal. Each individual was granted a maximum of 6 trials to succeed in this phase. An animal was considered to have learned the detour test if it successfully reached its conspecifics in 2 consecutive trials, marking the completion of the training phase. Animals that did not meet the learning criterion were not subjected to further testing and were attributed a maximum learning latency of 360 s.

**Figure 4:**
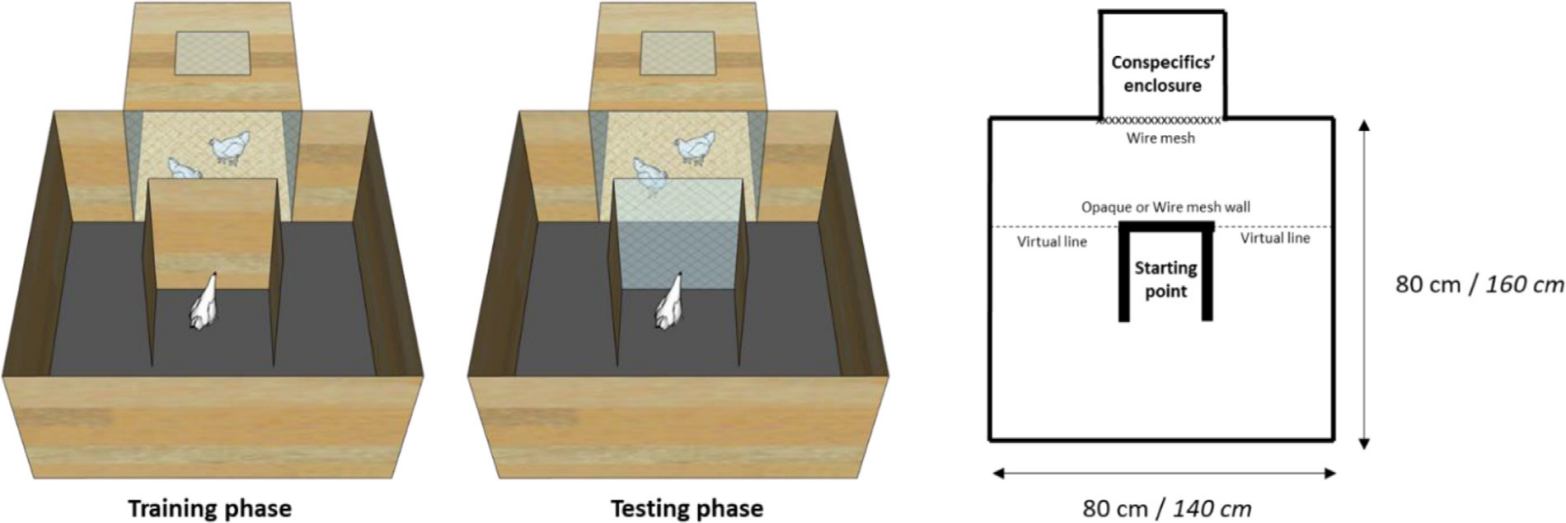
Schematic representation of the arena used for the detour test in chicks (from 16 to 20 d of age), before range access, and pullets (at 98 d), after range access. The squared arena consisted of a U-shaped obstacle in which its central wall was either opaque (during training) or substituted by a wire mesh (during testing). The arena was connected to an enclosure with two conspecifics and visual contact between the tested bird and its conspecifics was possible and assumed from the moment the bird crossed the virtual line. Tested animals first learned to detour the opaque obstacle and then were exposed to the wire-meshed obstacle. Measurements in italics refer to the tests performed after range access.

For individuals that successfully learned the detour test during the previous training phase, the test phase followed: these individuals were briefly removed from the arena and placed in an isolated compartment for 2 min. Subsequently, they were returned to the arena, with 1 alteration—this time, the opaque central wall of the U-shaped starting point was replaced by a wire mesh. This modification allowed the tested bird to observe its conspecifics from the starting point. The bird was then provided with a maximum of 5 min to reach its conspecifics by applying the same detour they have learned in the previous training phase (Figure 4). The latency (in seconds) for the individual to reach the zone close to its conspecifics by crossing the virtual line (with at least 50% of its body) was measured.

#### Multivariate Test

In previous studies, different behaviors expressed before range access were seen to correlate with range use, either negatively as was the case of social motivation (broilers: Ferreira et al., 2020a), or positively as was the case of foraging/exploration (broilers: Ferreira et al., 2021; Ferreira et al., 2022; Bonnefous et al., 2023); (laying hens: Kolakshyapati et al., 2020). To further investigate these relationships, here, we applied a multivariate test aimed to measure the expression of several animal behavioral traits, indirectly, such as its degree of social motivation, exploration and boldness from its use of the arena, or directly, such as foraging (Karlsson et al., 2011; Wirén and Jensen, 2011; Zidar et al., 2018; Bonnefous et al., 2023).

**Figure 5:**
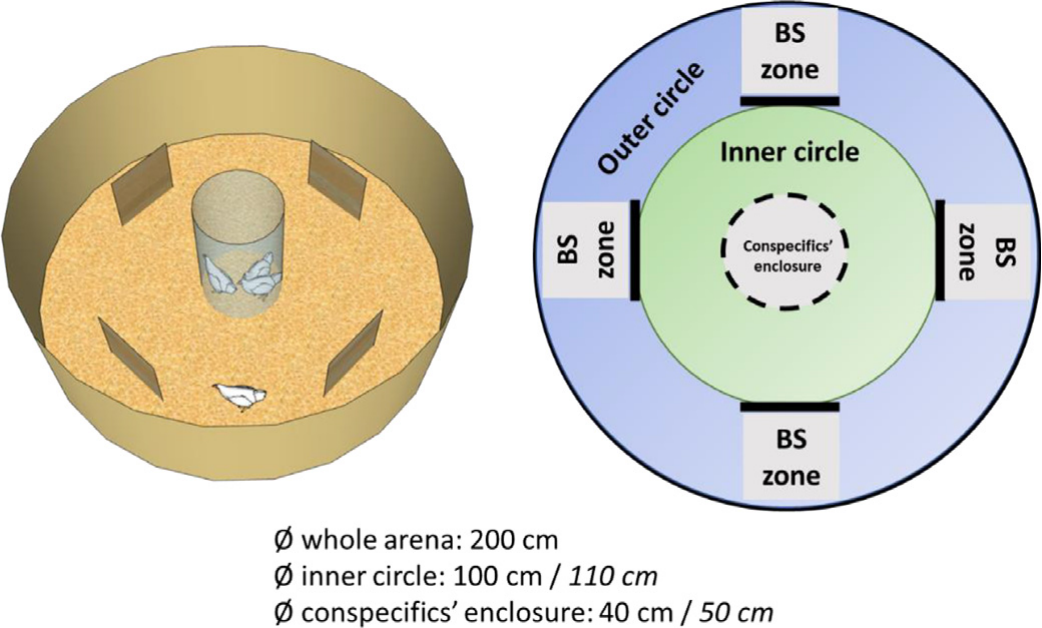
A schematic representation of the arena used for the multivariate test, conducted with chicks (from 37 to 39 d of age), before range access, and pullets (at 108 days), after range access. At the center of the arena, 3 conspecifics were accommodated in a circular wired zone. Four identical screens were placed to form an incomplete circle. The tested bird could access 3 virtually different zones: the inner circle, near its conspecifics; the outer circle, in visual contact but distant from its conspecifics; and the zones behind the screen (BS zone), where it was distanced from its conspecifics and lacked visual contact with them. All birds initiated the test from the same position, on the delimitation between the inner circle and the outer circle (so as not to favor one zone over the other). Measurements in italics pertain to the tests conducted after range access.

This test was conducted when the animals were between 37 to 39 d of age (before range access) and then again at 108 d of age (after range access). The test arena, constructed from flexible plastic, took on a circular shape (Ø: 200 cm x 50 cm) and featured a central wire area designed to accommodate 3 conspecifics (Ø before range access: 40 cm; Ø after range access: 50 cm). These conspecifics were selected at random and changed every 5 individuals tested. Four identical screens (before range access: 36 × 36 cm; after range access: 42 × 42 cm) were strategically positioned in the arena, forming an incomplete circle (Ø before range access: 100 cm; Ø after range access: 110 cm, Figure 5). To encourage foraging and exploration, the arena floor was composed of wood shavings. At the onset of the test, the tested individual was placed in the central compartment, in the presence of 3 conspecifics, for a 3-min period to acclimatize to the arena surroundings. Following this habituation phase, the tested individual was positioned at a predetermined starting point and given 5 minutes to explore the test arena. The arena was partitioned into 3 virtual zones to assess how birds utilized the space during the test. In the “inner circle” zone, the bird was considered to be in close proximity to its conspecifics, while in the “outer circle” zone, it was no longer in direct proximity to its conspecifics but still maintained visual contact with them. Finally, if the bird moved to the “behind a screen” zone, it was completely out of sight of its conspecifics (Figure 5).

Five different variables were measured, including the time spent in each of the 3 zones (in seconds), the number of zone changes (i.e., how frequently an individual moved from one zone to another), and the total time spent foraging (i.e., the animal pecked or scratched the ground with its paws in a standing position, measured in seconds). An individual was considered to be within a specific zone when at least 50% of its body was situated in that zone.

#### Ranging Behavior Measures and Classification of High and Low Rangers

**Figure 6:**
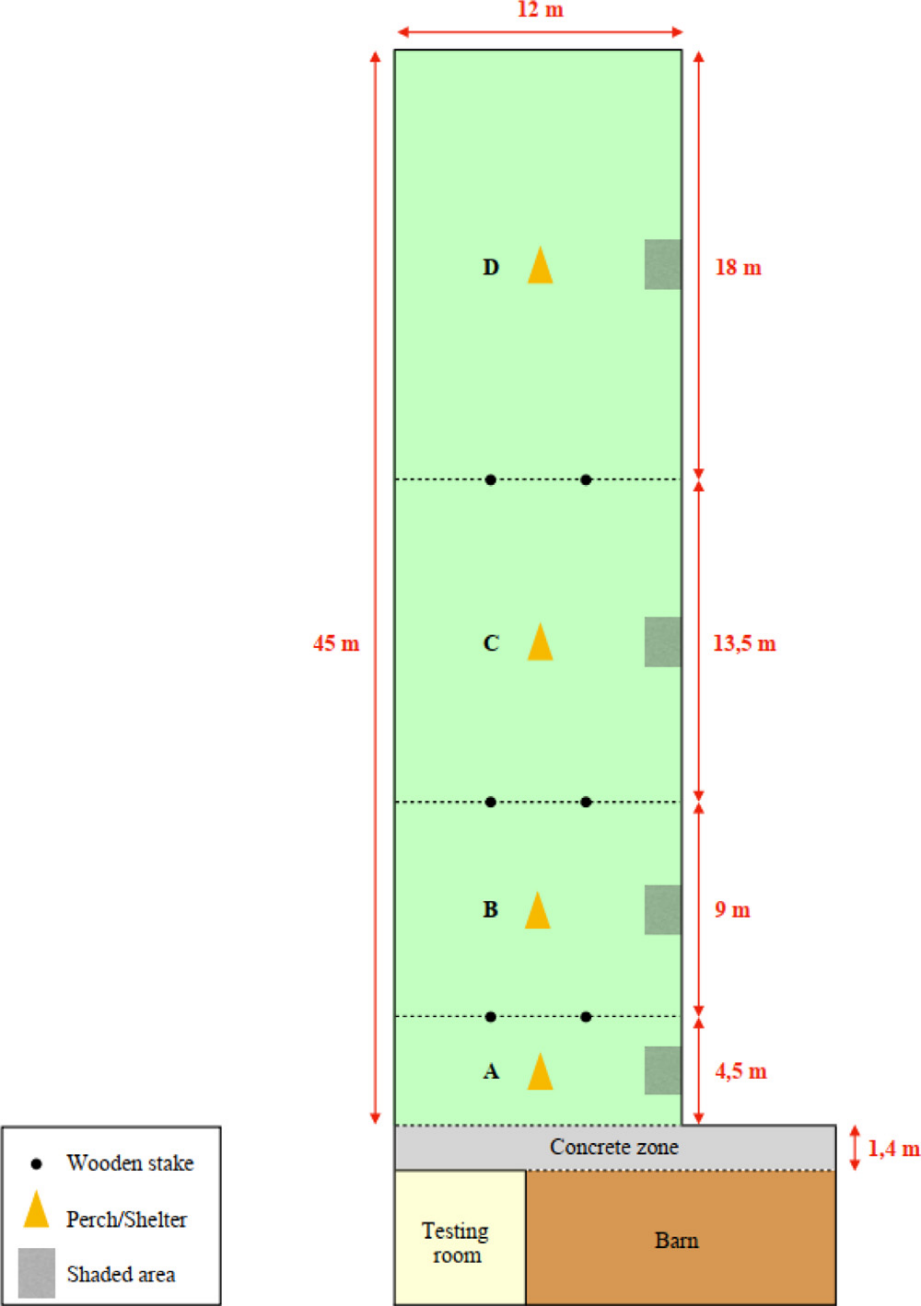
A schematic representation of the range, virtually divided into 4 different zones, demarcated with multiple wooden stakes (black dots). Zones A–D increased in length gradually (A = 4.5 m, B = 9 m, C = 13.5 m, and D = 18 m). This range division enabled a greater distinction between chickens that remained near the barn and those that ventured further into the range. The range was equipped with perches, shelters, and shaded areas that were equally distributed among the different zones to encourage birds to visit and explore the range.

The birds were allowed to access the range at 71 d of age. The outdoor range encompassed a concrete space in front of the barn and 4 distinct zones, denoted as A, B, C, and D, arranged in order from closest to farthest from the barn. These zones were demarcated with wooden stakes, each progressively longer, facilitating the distinction of inter-individual variability, since some individuals are known to remain in proximity to the barn (Hegelund et al., 2005; Ferreira et al., 2019). These zones were also uniformly equipped with a perch/shelter and a shaded area, intended to encourage birds to use the range evenly (Figure 6).

To assess the birds’ individual use of the range, we adopted the same procedures as described by (Ferreira et al., 2021; Ferreira et al., 2022; Bonnefous et al., 2023). In summary, on each scan day, an observer quietly entered the range. After a brief period of remaining stationary (approximately 10 min) to allow the birds to acclimate to her presence and resume their normal behaviors, she calmly walked through the range and initiated the scan sampling process. This involved recording the identity and location of the birds present in the range. Scan measurements started 3 d after the individuals were allowed access to the range, beginning at 74 d of age and concluding at 91 d of age during the summer season, spanning from mid-June to early July. Scans were carried out over 7 nonconsecutive days, with intervals of 1 to 4 d between each scanning session, aiming to distribute the observation days evenly and minimize potential weather-related biases in assessing the birds' range use. Rainy days were intentionally avoided to ensure the standardization of data collection. Observations were made 8 times a day at regular intervals, specifically at 9:00, 10:00, 11:00, 12:00, 14:00, 15:00, 16:00, and 17:00, resulting in a total of 56 scans. In the event that an individual was not within any of the range zones during the scan, it was deduced to be inside the barn.

Based on the collected data, an individual distance index (**DI**) was computed (Ferreira et al., 2019). This index considered the frequency of an individual's presence in a specific range zone, multiplied by half the length of that zone, added to the total length of previously traversed zones (applicable to zones A, B, C, or D). For instance, if a particular individual was observed in zone B, we calculated that it had covered 1.4 meters of the concrete zone plus 4.5 meters of zone A (total length), and another 4.5 meters of zone B (half-length).

The distance index was then determined using the following formula: Distance index **=** (number of times seen in the concrete zone * 0.7) + (number of times seen in zone A * 3.65) + (number of times seen in zone B * 10.4) + (number of times seen in zone C * 21.65) + (number of times seen in zone D * 37.4). A high distance index indicated a high range use.

From our initial sample of 100 individuals, birds situated at the opposite ends of the distance index spectrum were categorized as either high rangers (**HR**, the top 15% with the highest distance index, n = 15) or low rangers (**LR**, the bottom 15% with the lowest distance index, n = 15). High rangers denoted individuals that frequently ventured to the range, covering greater distances from the barn, while low rangers represented birds that exhibited a preference for remaining within or near the barn. These 2 groups of birds proceeded to the second round of testing, which included the cognitive bias, detour test, and multivariate test, as previously described.

### Statistics

Statistical analyses were conducted in R 4.3.1. (Team, 2013) and IBM SPSS 25. They were structured in 2 main parts. Initially, we conducted an investigation of all the tested chicks across the 3 tests performed before range access, assessing their correlations with range use. Subsequently, we narrowed our focus on the outliers within the range use continuum—namely, the high rangers and low rangers—and investigated their respective measures both before and after range use. Since birds were expected to gradually increase their range use over time as they became more familiar with their surroundings (Grigor et al., 1995a; 1995b; Pettersson et al., 2016; Ferreira et al., 2022), we divided the range use variables (number of range visits and individual distance indexes) into 3 distinct periods comprising the first 3 scan days (Period 1, P1 – D 74, 77, and 79 d old), the subsequent 4 scan days (Period 2, P2 – 81, 84, 88, and 91 d old), and, ultimately, the combination of data from all days (Total range use – from 74 to 91 d old).

To assess whether our cognitive bias test produced results consistent with previous studies employing the same testing paradigm (Salmeto et al., 2011; Hedlund et al., 2021, 2022), we initially conducted an analysis of the first cognitive bias test alone, before range access, on the latency birds took to reach the virtual line. This analysis considered the influence of the stimuli themselves (mirror, chick/pullet, morph, and owl) and the presentation order of stimuli (either chick/pullet or owl during the second trial) as fixed effects. These factors and their interactions were examined using a linear mixed model (using the ‘lmerTest’ package) (Kuznetsova et al., 2017). We accounted for repeated measurements by considering individual ID as a random factor. Latency to reach the virtual line was square-root/log transformed to approach residual normality. We checked models’ assumptions using the DHARMa package (Hartig, 2022). Only significant interactions and fixed effects were kept in the final model. When necessary, we performed post-hoc ANOVA comparisons of the estimated marginal means using the “emmeans” package, with Tukey adjustment to address multiple comparisons (Lenth et al., 2018).

After this initial assessment, a Spearman rank correlation was carried out between the latency to reach the virtual line for each of the 4 stimuli and the individual number of range visits and to individual distance indices (P1, P2, and Total range use). Finally, for the comparison between extremes (high vs. low rangers), following the same steps as the above-mentioned initial analysis, we ran a second linear mixed model with stimuli, ranging level (high or low ranger), the presentation order of stimuli (either chick/pullet or owl during the second trial), and time of test (before and after range access), as well as their interactions, as fixed factors.

The relationships between the latency to learn and complete the detour test and the range use variables (such as the number of range visits and individual distance indices in P1, P2, and Total range use) were explored through non-parametric Spearman rank correlations. Given that a majority of individuals did not meet the learning criterion in the second round of the detour test after range access and were consequently excluded from testing (see more details below), a survival analysis (“survival” package, Therneau and Grambsch, 2000) comparing high and low rangers was conducted solely for the first round (before range access).

Finally, the relationships between the variables recorded during the first round of the multivariate test (the time spent in each of the three zones (inner circle, outer circle, and behind the screens), the number of zone changes, and the total time spent foraging) and range use variables were also investigated through non-parametric Spearman rank correlations. Similar to the cognitive bias test analyses, to compare high and low rangers, we ran a model including testing round (before and after range access), ranging level (high or low ranger), as well as their interactions, as fixed factors. We accounted for repeated measurements by considering individual ID as a random factor. Variables were transformed, when necessary, to approach residual normality. We checked models’ assumptions using the DHARMa package (Hartig, 2022). Only significant interactions and fixed effects were kept in the final model.

During the experiments, 8 individuals were excluded from the study for various reasons (death of the animal, injuries, etc.), reducing the sample size from 100 to 92 birds. Results are presented as raw means ± SD.

## RESULTS

### Cognitive Bias

**Figure 7:**
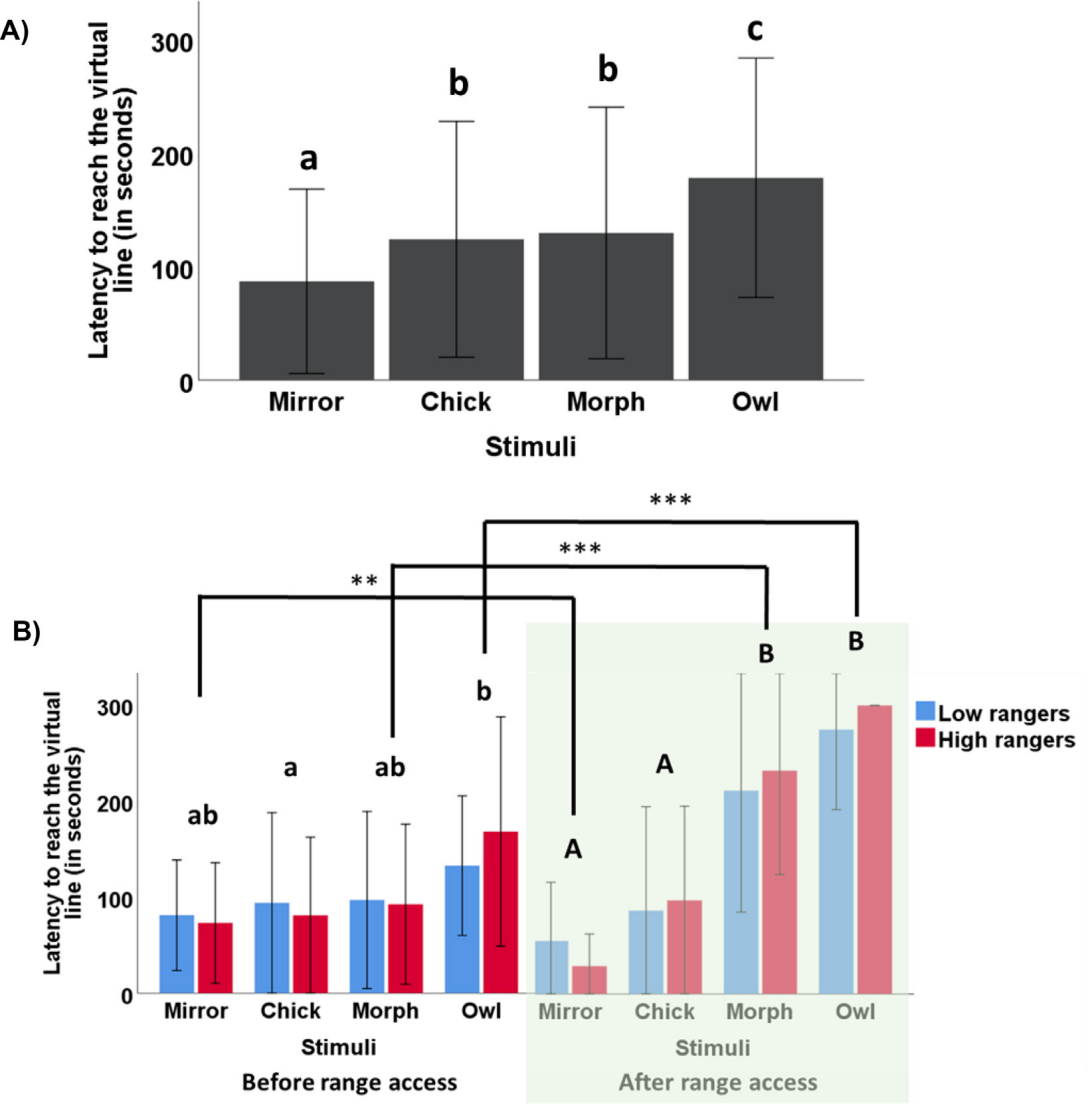
Mean latency to reach the virtual line (± standard deviation) in the cognitive judgement bias test when birds were exposed to a mirror, chick/pullet, morph, or owl stimulus. (A) Initial analysis on the entire sample of 83 chicks successfully tested before range access (9 to 13 d of age). (B) Subsequently analysis on 24 birds categorized as either low (blue bars) or high rangers (red bars), retested after range access (94–95 d of age). In addition to comparing birds on the extremes of the range use continuum, this analysis provided insight into the potential changes in behavior over time. Lowercase letters indicate significant differences between stimuli before range access. Uppercase letters indicate significant differences between stimuli after range access. Asterisks indicate significant differences within a stimulus, comparing before and after range access. **p < 0.01, ***p < 0.001.

Out of the initial group of 92 chicks, 6 chicks did not successfully reach the virtual line during their first trial, when the mirror was used as a stimulus. The remaining 86 individuals were subjected to all 4 stimuli. The latency to reach the virtual line was significantly influenced by the stimulus presented in the arena (LMM, F (3,255) = 19.29, *p* < 0.001), but not by the stimuli order presentation (LMM, F (1,84) = 0.68, *p* = 0.41). Post-hoc analysis showed significant differences in latency between each stimulus (all *p* < 0.05, n = 86), with the exception of the comparison between the chick and morph stimuli (*p* = 0.99, n = 86). The presence of the mirror resulted in the lowest latency (87.01 ± 81.04 s), while chick and morph latencies were intermediate (123.9 ± 103.72 sec and 129.45 ± 110.54 sec, respectively). On the other hand, the owl stimulus generated the highest latency (178 ± 105.2 s, Figure 7A).

None of the Spearman correlations between the latencies to reach the stimuli and range use variables were significant (all *p* > 0.05, n = 86, Table 1). However, there was a trend for the morph stimulus to correlate negatively with the range use variables of the first period (P1) of range observations (range visits: rho = -0.19, *p* = 0.07, and distance index: rho = -0.18, *p* = 0.09, Table 1).

**Table 1:** Rank correlations between variables recorded during chicks’ individual tests (cognitive bias test, detour test, and multivariate) and the range use variables (number of range visits and individual distance indexes) over 3 distinct periods comprising the first 3 scan days (Range Use 1), the subsequent 4 scan days (range use 2), and, ultimately, the combination of data from all days (total range use). Green-highlighted cells denote statistically significant correlations (*p* < 0.05), whereas yellow-highlighted cells indicate trends (*p* < 0.1).

The rerun model including only high and low rangers tested both before and after range access revealed a significant influence of stimuli (LMM, F[3,160.28] = 23.82, *p* < 0.001), time of test (LMM, F[1,160.28] = 5.78, *p* < 0.001), and a significant interaction between stimuli and time of test (LMM, F[3,160.28] = 9.3, *p* < 0.001) on the animals’ latency to reach the virtual line. However, there was no discernible influence observed from testing order (LMM, F[1,165.84] = 0.28, *p* = 0.59) or ranging level (LMM, F[1,22.87] = 0, *p* = 0.97). In contrast to the previous analysis on chicks before range access, in this specific subsample, and considering only the stimuli effect, the latency to reach the virtual line in the presence of the mirror and pullet stimuli did not exhibit a significant difference (*p* = 0.49). However, it increased significantly and differed for all other stimulus comparisons (mirror = chick < morph < owl, all *p* < 0.05). Moreover, when examining the post-hoc analysis of the interaction between stimuli and time of test, we observed variations in the birds' responses to stimuli between the first and second test rounds (conducted before and after range access, respectively). Prior to range access, this subset of birds exhibited more uniform behavior towards all stimuli, albeit with the exception of a significant difference between chick and owl (*p* = 0.04), and a tendency to differ between mirror and owl (*p* = 0.09). However, following range access, distinct patterns emerged: the mirror-chick pair generated similar low latencies, while the morph-owl pair resulted in high latencies. Additionally, comparison of stimuli latencies between periods revealed that birds approached the mirror more quickly in tests following range access compared to the corresponding trials before range access (*p* = 0.01). Conversely, the reverse trend was observed for the morph and owl trials: birds took longer to approach these stimuli when tested after range access compared to tests conducted before range access (*p* < 0.001, see Figure 7B).

### Detour Test

No significant correlation was found between the range use variables and the latency to learn to detour the obstacle during the training phase (all *p* > 0.05, n = 92, Table 1). Among the 79 individuals who successfully completed the training and were tested, we identified negative correlations between the time taken to complete the test and the range visits and individual distance index during P1 (rho = -0.23; *p* = 0.036, n = 79 and rho = -0.25; *p* = 0.023, n = 79, respectively). This indicates that chicks that took longer to detour the obstacle were less inclined to use the range as soon as it was available (first days). No such correlations were found for the other periods (P2 or Total Range Use, Table 1).

**Figure 8:**
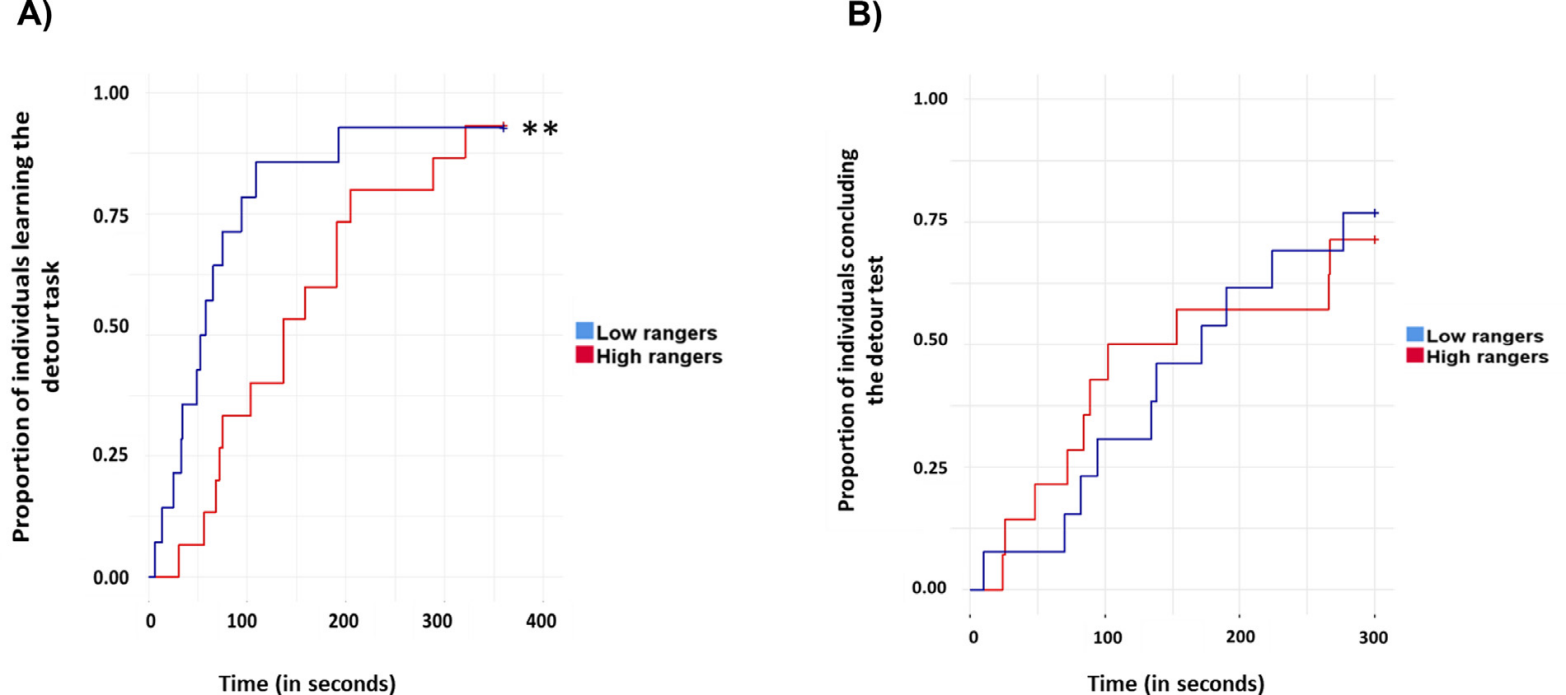
Birds’ performance during the detour task conducted prior to range access (between 16 and 20 d of age). (A) Learning latency of birds during the training phase of the detour task. Each bird underwent a maximum of 6 trials, each lasting 60 s. Successful training was achieved if a bird successfully detoured the obstacle in two consecutive trials, marking their progression to the final test. (B) Individuals that successfully learned the detour during the training phase were subjected to the testing phase and were allowed a maximum of 5 minutes to detour the obstacle and reach their conspecifics by applying the same detour they have previously learned. Asterisks indicate significant differences between high and low rangers. (n_Low rangers_ = 15, n_High rangers_ = 14). ** *p* < 0.01.

Following range access, in the second round of testing, 23 out of 29 individuals exhibited maximum latency values during detour training, indicating a notably low social motivation to rejoin their conspecifics. Only 6 individuals proceeded to the testing phase, comprising 4 high rangers and 2 low rangers. Consequently, comparisons between high and low rangers were exclusively conducted for the first testing round (before range access) using a survival analysis. These analyses unveiled a significantly shorter learning latency for low rangers compared to high rangers during the training phase (83.5 ± 92.59 s and 159.66 ± 100.6 s, respectively; χ2 = 7.34, df = 1, *p* = 0.006, n_Low rangers_ = 15, n_High rangers_ = 14, Figure 8). However, no significant differences were observed during the test phase (χ2 = 0.12, df = 1, *p* = 0.72, n_Low rangers_ = 14, n_High rangers_ = 13).

### Multivariate Test

Regarding the 92 chicks subjected to the multivariate test, we found negative and significative correlations between the range visits and the distance index during P1 and the time birds spent in the inner circle (rho = -0.23, *p* = 0.02 and rho = -0.21, *p* = 0.04, respectively), while these correlations tended towards significance when considering the total number of range visits (rho = -0.18, *p* = 0.07). A different pattern emerged for the relationship between our 2 range use variables and the time spent in the outer circle (P1/Range visits: rho = 0.3, *p* = 0.004; P1/Distance index: rho = 0.27, *p* = 0.009; Total Range use/Range visits: rho = 0.24, *p* = 0.01; Total Range use/Distance index: rho = 0.22, *p* = 0.03) and the number of zone changes (P1/Range visits: rho = 0.26, *p* = 0.01; P1/Distance index: rho = 0.25, *p* = 0.01; Total Range use/Range visits: rho = 0.23, *p* = 0.02; Total Range use/Distance index: rho = 0.22, *p* = 0.04). No correlations were found between any of the test variables and range use during P2. Similarly, there were no observed correlations between any range use variables (in any period or the total range use) and the variables measuring time spent behind a screen, nor time spent foraging (Table 1).

**Figure 9:**
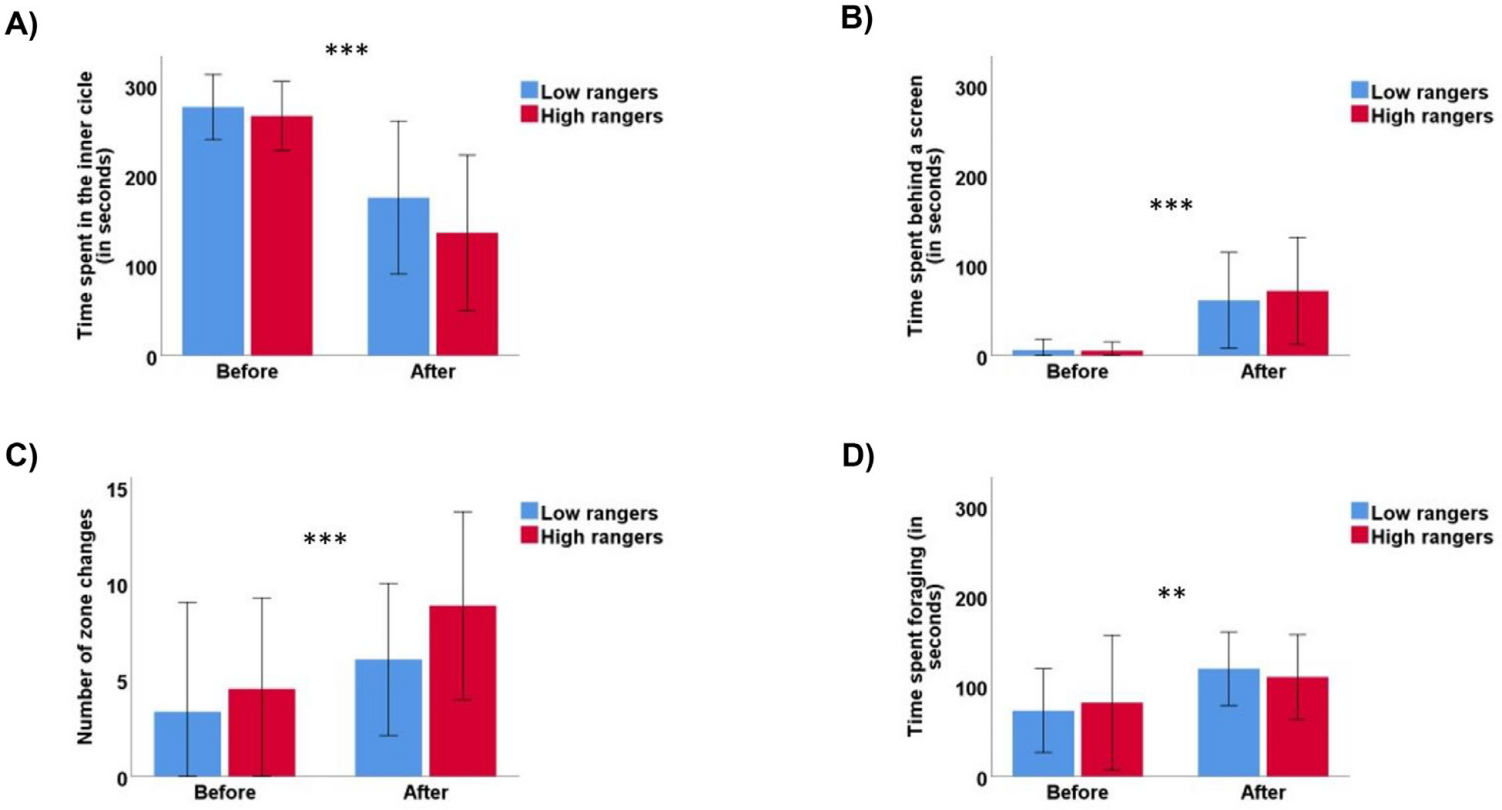
Variables recorded during the multivariate test carried out before (37–39 d of age) and after (108 d of age) range access: (A) time spent in the inner circle (seconds); (B) time spent behind a screen (seconds); (C) number of zone changes; (D) time spent foraging (seconds). n_low rangers_ = 14, n_high rangers_ = 15. Asterisks indicate significant within-variable differences before and after range access. *** *p* < 0.001; ** *p* < 0.01.

The analysis of our subsample, tested both before and after range access, revealed significant differences across various testing periods for all variables. However, distinctions between high and low rangers were limited. In the multivariate test conducted after range access, compared to their initial session, birds exhibited a decrease in time spent in the inner circle of the arena (squared-root transformed, F[1,28] = 45.47, *p* < 0.001, Figure 9A). Conversely, there was an increase in time spent in the outer circle (F[1,28] = 30.62, *p* < 0.001) or behind a screen (squared-root transformed, F[1,28] = 49.72, *p* < 0.001). Additionally, they displayed increased zone changes (F[1,28] = 12.20, *p* = 0.001) and an increased propensity for foraging (F[1,28] = 8.39, *p* = 0.007). High rangers showed a tendency to spend more time in the outer circle compared to low rangers (59.96 ± 49.68 s and 40.21 ± 44.48 s, respectively; F[1,27] = 3.64, *p* = 0.06, n_Low rangers_ = 14, n_High rangers_ = 15), with no other significant differences found between the 2 groups (all *p* > 0.05).

## DISCUSSION

The aim of the present study was to gain a deeper understanding of the relationship between range use, behavior and cognition in White Leghorn laying hens reared in a free-range system. More specifically, we first aimed to assess whether certain behavioral and cognitive aspects of the young animals, prior to their access to the range, are indicative of their future range use, when birds are older. Subsequently, focusing on the individuals with the highest and lowest range use (high and low rangers, respectively), we aimed to explore how their behavior and cognition change over time and how these aspects relate to their respective range use. To this end, three individual tests (the cognitive bias test, the detour test and the multivariate test) were carried out both before and after birds were granted access to the range. Individual range use was assessed by tracking the number of times a bird visited the range and its distance from the barn in the weeks following the availability of the range. Overall, our results showed that, while birds perceived and reacted as expected to the 4 different stimuli presented during the cognitive bias test, only the ambiguous cue (i.e., the morph of a chick/pullet and an owl) demonstrated a weak tendency to correlate negatively with range use. Additionally, we observed that low rangers mastered the detour test more quickly during the training phase (with an opaque barrier) compared to high rangers. This outcome is supported by the negative correlations between range use and the time taken to complete the detour test during the testing phase (with a wire-meshed barrier). Finally, the multivariate test unveiled potential associations between birds' exploratory and sociability tendencies and their range use. Individuals that ventured farthest from their conspecifics and moved more within the arena were also those who used the range more. Significant behavioral changes were noted between the 2 testing rounds, conducted before and after range access. This observation underscores the importance of exercising greater caution in considering the developmental trajectory of birds over time and its potential impact on test outcomes.

The cognitive bias test showed that the latency to reach the virtual line differed according to the stimulus positioned in the arena: there was a gradual increase in the latency to approach the different stimuli, with the birds taking less time to approach the mirror, and a long time to approach the owl stimulus, the latter being indicative of greater vigilance. Latencies to approach the chick/pullet and morph stimuli were intermediate. These results are similar to those obtained in previous studies (Salmeto et al., 2011; Hedlund et al., 2021, 2022), and corroborates that chicks are able to perceive the stimuli differently and adapt their behavior accordingly.

Our initial hypothesis regarding the existence of correlations between optimism/pessimism perception and range use was not validated, consistent with previous findings. These findings showed that hens’ visits to ambiguous arms in an 8-arm radial maze did not correlate with range use but are instead related to environmental enrichment (Taylor et al., 2023). It is noteworthy that an intriguing trend emerged. More precisely, birds with a prolonged latency in approaching the morph (i.e., the ambiguous stimulus) demonstrated reduced range use during the initial days of observation compared to birds that approached the morph more swiftly. However, this trend was not consistent during the later days of observation, nor did it hold true for the total range use (sum of the first 3 d and the last 4 d of range use observation). It is possible that, during the initial days of accessing the range, birds mainly rely on their own evaluations (and own cognitive biases) of the environment and, over time, with more and more birds visiting the range as days progress, this pattern may change over time, and social influences may overcome individual perceptions. Future studies should investigate these speculations further. Overall, these findings imply that the early inter-individual differences in chick behavior may be intricately tied to their cognitive biases. This connection, in turn, influences how they interact with their environment, aligning with observations in other species (Cussen and Mench, 2014; Asher et al., 2016; Lecorps et al., 2018).

With regard to the detour test, our initial predictions were only partially confirmed: before range access, chicks that took a longer time in completing the final test were also those that used the range less, while after access to the range, low-ranging individuals exhibited a significantly shorter latency in learning the detour test during the training phase compared to their high-ranging counterparts. Our results partially align with those of Campbell et al. (2018b), who observed that high-ranging pullets demonstrated a faster learning curve in locating a food reward in a T-maze compared to their low-ranging conspecifics, but also align with the results of Ferreira et al., (2019; 2020c), who reported contrasting findings for slow-growing broiler chickens: their research indicated that low-ranging chickens not only possessed superior spatial memory abilities but also displayed enhanced cognitive flexibility, adapting more effectively to changing situations. These results may stem from the more fearful personality of low-ranging individuals (Campbell et al., 2016, 2019a, 2019b), prompting increased attention to their environment and facilitating quicker learning compared to their high-ranging counterparts (Sih and Del Giudice, 2012). It is important to bear in mind that different studies used animals selected for different purposes, such as free-range laying hens and broiler chickens. Additionally, the criteria for classifying high and low rangers may differ, as well as the age the subjects were tested. For instance, Campbell et al., (2018b) measured pullets’ range use between 22 to 36 wk of age, while the current study measured range use of birds aged 10 to 13 wk. Hence, exercising caution is imperative when interpreting and comparing findings across different studies.

An alternative explanation for the increased performance of low-ranging birds in the detour test could be linked to their increased sociability. Since the test heavily relies on social motivation, it is plausible that these individuals, owing to their elevated sociability levels, are more inclined to join their conspecifics. Consequently, they may have been quicker to conclude the test, without necessarily showcasing superior cognitive abilities than their high-ranging counterparts. In free-range systems, individuals that remain in or close to the barn encounter a higher density of conspecifics (number of animals per m^2^), suggesting that low rangers might indeed be more sociable (Ferreira et al., 2020a). Indeed, social isolation on the range may be more frequent given that the density decreases with distance from the barn (Chielo et al., 2016; Taylor et al., 2020).

The outcomes of the multivariate test lend support to the hypothesis that low-ranging birds exhibit heightened sociability: chicks remaining in proximity to their conspecifics displayed a reduced inclination to use the range. Conversely, those displaying more exploratory behaviors in the arena—whether through visiting multiple zones or distancing from conspecifics—also exhibit increased exploration of the range later in life. These findings align with our prior research on free-ranging broiler chickens, suggesting the presence of within-individual behavioral consistency over time, akin to personality traits (Ferreira et al., 2021a; Ferreira et al., 2022). Interestingly, we did not find any relationship between foraging behavior and range use, as previous seen for broilers (Ferreira et al., 2021a; Ferreira et al., 2022). These contrasting results reinforce that behavioral/cognitive predictors of range use may diverge between laying hens and broilers, but also among distinct strains (Wurtz et al., 2022; Bonnefous et al., 2023), and that these differences need to be further investigated to understand how these traits shape how chickens perceive, interpret, and interact with their environment.

In the analyses concerning only high and low rangers, discernible behavioral and cognitive shifts emerged when comparing testing sessions before and after range access. Notably, in the cognitive bias test, birds displayed heightened vigilance towards the morph and owl stimuli after the range access (when animals are older), compared to the test carried out before the range access (when animals are younger). Additionally, during the detour test subsequent to range access, an apparent lack of social motivation impeded the birds' progress in the testing phase. This observation is substantiated by changes observed in the multivariate test sessions. Overall, the animals displayed a decrease in sociability, as evidenced by reduced time spent in the inner circle, coupled with an increase in exploratory behaviors (more instances of being without visual contact with conspecifics, greater frequency of zone changes, and increased time devoted to foraging). Future longitudinal studies comparing animals with and without range access, as well as those that underwent testing and those that did not, could offer a more nuanced understanding by disentangling the potential effects of age, range use, testing, and testing habituation (test/retest on the same individuals) on the variables of interest. To better tailor and fortify the robustness of these behavioral/cognitive tests, as well as to mitigate potential methodological artifacts, it is crucial to remain mindful that these factors could exert a significant influence on testing outcomes.

Taken together, our results put into evidence the intricate relationships existing between cognitive and behavioral traits in young laying hen chicks, significantly influencing their subsequent range use. In particular, a pronounced social motivation appears to limit the animals' range use, as they tend to stay in close proximity with the majority of their conspecifics within the barn. Conversely, early individual exploratory tendencies correlate with an increased propensity for range use. Consequently, the extent of range use emerges as a complex balance between an individual's motivation to stay near conspecifics and its drive to explore the environment. These early behavioral indicators could serve as predictors of an animal's level of range use. Future studies should delve into the intricate connections between cognition and range use, as preliminary evidence suggests that individual spatial abilities and cognitive biases may play a role in determining an individual's willingness to explore the range. Accurately identifying and characterizing the behavioral and cognitive differences within a flock, and understanding their relationships to range use, can allow us to more effectively address the individual needs of these animals in the future (Ferreira et al., 2021a), with the potential to not only increase their range use but also enhance their overall welfare.
